# Apparent Oxygen Uphill Diffusion in La_0.8_Sr_0.2_MnO_3_ Thin Films upon Cathodic Polarization

**DOI:** 10.1002/celc.201500167

**Published:** 2015-07-21

**Authors:** Tobias M. Huber, Edvinas Navickas, Gernot Friedbacher, Herbert Hutter, Jürgen Fleig

**Affiliations:** ^1^ Institute of Chemical Technologies and AnalyticsVienna University of TechnologyGetreidemarkt 9ViennaA-1060Austria

**Keywords:** cathodic bias, grain boundaries, mass spectrometry, thin films, tracer diffusion

## Abstract

The impact of cathodic bias on oxygen transport in La_0.8_Sr_0.2_MnO_3_ (LSM) thin films was investigated. Columnar‐grown LSM thin films with different microstructures were deposited by pulsed laser deposition. ^18^O tracer experiments were performed on thin film microelectrodes with an applied cathodic bias of −300 or −450 mV, and the microelectrodes were subsequently analyzed by time‐of‐flight secondary ion mass spectrometry. The ^18^O concentration in the cathodically polarized LSM microelectrodes was strongly increased relative to that in the thermally annealed film (without bias). Most remarkable, however, was the appearance of a pronounced ^18^O fraction maximum in the center of the films. This strongly depended on the applied bias and on the microstructure of the LSM thin layers. The unusual shape of the ^18^O depth profiles was caused by a combination of Wagner–Hebb‐type stoichiometry polarization of the LSM bulk, fast grain boundary transport and voltage‐induced modification of the oxygen incorporation kinetics,

## 1 Introduction

La_0.8_Sr_0.2_MnO_3_ (LSM) and similar perovskite‐type materials are widely investigated for solid oxide fuel cell cathode applications.^1^ Owing to its low ionic conductivity, LSM is often considered to be a three‐phase boundary active material,1c and the bulk path, that is, oxygen reduction with ion transport through LSM, is relevant only in thin films.^2^ However, the oxygen reduction reaction at the surface of LSM and oxygen diffusion in LSM can be varied by an applied cathodic bias.2a, 3 The bias dependence of oxygen diffusion is due to stoichiometry changes in LSM upon polarization caused by a modified chemical potential of oxygen. Higher oxygen vacancy concentrations result and can improve the electrochemical performance of LSM electrodes. Therefore, the bulk path of oxygen reduction may be highly important not only in thin films but also in polarized porous LSM cathodes.^4^ Simulations of the relevance of the bulk path in polarized LSM electrodes are presented in Ref. 4b. An applied cathodic bias further affects the oxygen incorporation rate at the surface, though details of these changes and of the oxygen incorporation mechanism in LSM are not yet well understood. Additional experiments on LSM electrodes under operating conditions are therefore needed to obtain a clear picture of the kinetics of oxygen reduction occurring through the bulk path. Thin films are particularly useful in this respect, due to the enhanced relevance of the bulk path, their simple geometry, and the accessibility of the surface to surface analytical tools.

^18^O tracer diffusion and subsequent secondary ion mass spectrometry (SIMS) analysis is a powerful technique that allows oxygen exchange and oxygen ion diffusion to be probed and/or to visualize the active oxygen reduction sites.^5^ In previous studies, bias‐induced ^18^O incorporation experiments on LSM were successfully employed to qualitatively show the relevance of the bulk path for oxygen reduction.5b, 5c Recent contributions on thermal oxygen tracer incorporation into LSM thin films revealed further details on the mechanism of oxygen surface exchange and diffusion.5f, 6 It was shown that the grain boundaries play a major role and have diffusivities and surface exchange coefficients that are orders of magnitude higher than those of the grain bulk. Quantitative information on how oxygen incorporation and diffusion in LSM thin films is affected by a cathodic bias is still missing.

The goal of this work is to reveal the effect of a cathodic bias on oxygen incorporation and diffusion in LSM thin films by tracer experiments and subsequent depth profiling. To show the role of the thin film microstructure, layers with different grain widths were investigated. Bias‐driven isotope incorporation was performed on LSM microelectrodes with an applied cathodic bias of −300/−450 mV in the temperature range of 500 to 700 °C. The isotope depth profiles were measured by SIMS, and additional finite element modeling (FEM) revealed the high relevance of fast grain boundary transport but also the relevance of defect concentration gradients for oxygen reduction on LSM thin films under cathodic bias.

## 2 Results

**Figure 1:**
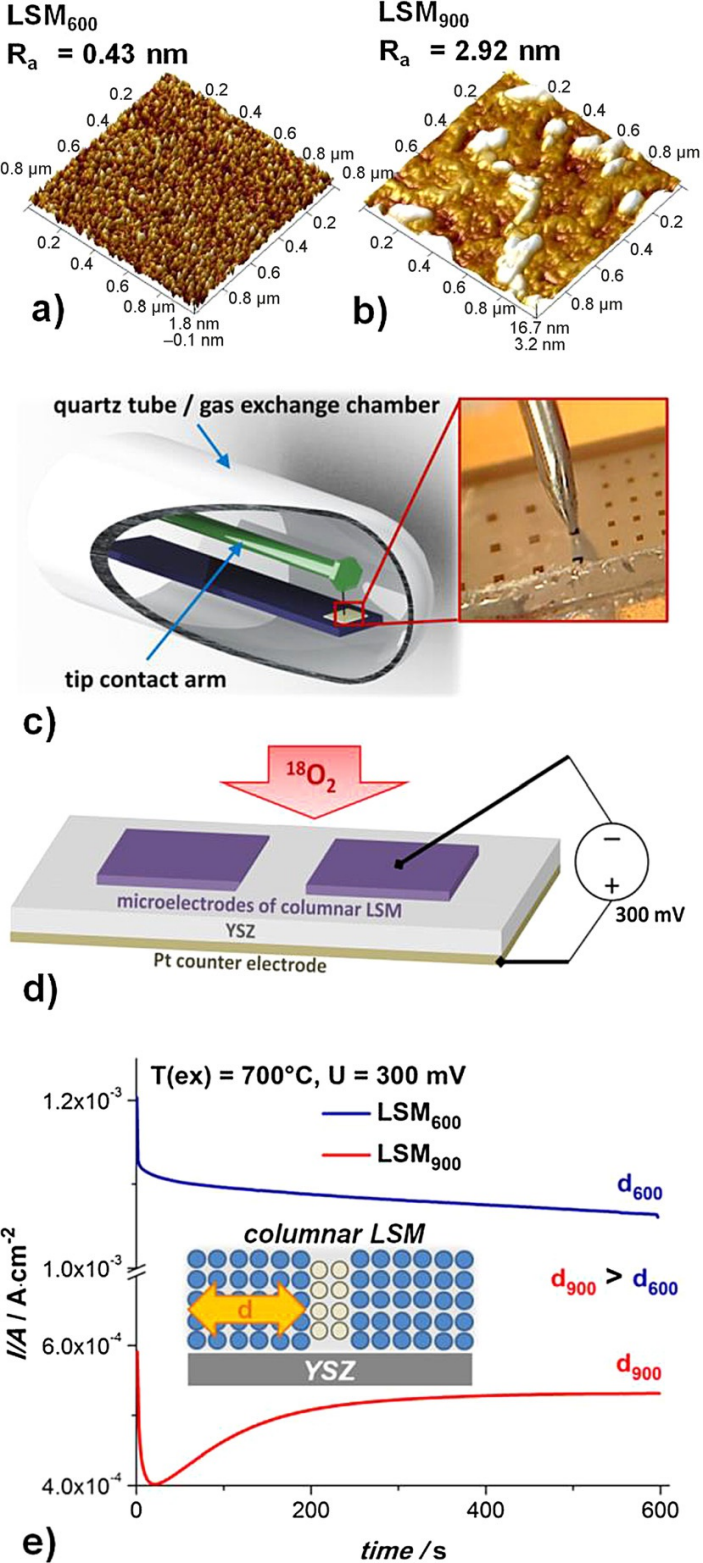
AFM micrographs (1 μm×1 μm) of LSM thin films prepared at a) 600 °C and b) 900 °C showing different microstructures. c) Sketch of a symmetrically heated measurement/gas‐exchange set up that includes a gas‐tight quartz chamber placed in a tube furnace, a contact arm with a contact needle, and a sample holder; a photograph of a contacted microelectrode is shown on the right‐hand side. d) Sketch of LSM microelectrodes on a YSZ substrate with electrical contact. e) The typical current response with an applied bias (−300 mV) obtained at 700 °C on microstructurally different layers (El7 deposited at 900 °C and El9 deposited at 600 °C).

Two sets of LSM samples were prepared at different deposition temperatures (600 and 900 °C), which led to different grain widths of the columnar grown films. As shown in Ref. 5f by using atomic force microscopy (AFM) and transmission electron microscopy (TEM), LSM layers deposited at 600 °C have grain diameters of about 30 nm and those deposited at 830 °C consist of grains that are roughly two times larger. Figure 1 a, b displays the surface topography of the LSM layers measured by AFM. The LSM layer deposited at 600 °C (LSM_600_) shows a surface topography with narrow and well‐defined grain width (≈30 nm, Figure 1 a). However, the LSM layer deposited at 900 °C (LSM_900_) has coarse surface features and much higher average surface roughness (Figure 1 b). This hindered determination of the exact grain width, but we concluded that this sample had a very different microstructure with grains larger than those found in LSM_600_.

Incorporation of the ^18^O isotope was performed on rectangular LSM microelectrodes with an applied cathodic bias *U* of −300 or −450 mV. In contrast to anodic voltages of similar magnitude, this cathodic polarization does not lead to visible morphological changes in the electrodes (no partial detachment).2a A microelectrode was electrically contacted in a symmetrically heated isotope exchange chamber (Figure 1 c, d), which helped to avoid inhomogeneous temperature distribution within the microelectrode.5a, 7 Immediately after ^18^O_2_ exposure, the contacted microelectrode was polarized and the dc current was monitored during the entire experiment. Each sample had numerous microelectrodes, and thus, unpolarized LSM was also exposed to ^18^O_2_, which allowed comparison with the thermal diffusion sample. An overview of all polarized electrodes is given in Table 1 with the deposition temperature (*T*_dep_), tracer exposure temperature (*T*_ex_), bias voltage *U*, cathodic overpotential *η*, current density found after 10 min of polarization, and tracer fraction at the LSM/yttria‐stabilized zirconia (YSZ) interface.

**Table 1:** The experimental parameters of the LSM microelectrodes. The measured current density after 600 s and the resulting ^18^O fraction at the LSM/YSZ interface are also given.

Electrode	*T*_dep_ [°C]	*T*_ex_ [°C]	Bias/*η* [mV]	*I*/*A* [A cm^−2^]	^18^O fraction in YSZ
El1	600	600	300/281	9.00×10^−4^	0.010
El2	600	700	300/265	8.33×10^−3^	0.057
El3	600	500	300/291	6.25×10^−5^	0.003
El4	600	500	450/368	6.25×10^−4^	0.008
El5	900	500	450/405	1.25×10^−4^	0.005
El6	900	600	450/430	1.12×10^−3^	0.016
El7	900	600	300/291	5.26×10^−4^	0.005
El8	900	700	300/291	3.10×10^−3^	0.015
El9	600	600	300/278	1.06×10^−3^	0.025

[a] *T*_ex_=tracer exposure temperature, *T*_dep_=LSM thin film deposition temperature, *η*=overpotential.

Table 1 indicates that, as expected, the measured current increases with measurement temperature and cathodic bias. Moreover, it was found that the LSM deposition temperature (*T*_dep_) also plays a significant role for the bias‐driven current. Typical current versus time graphs for an applied cathodic bias (−300 mV) are shown in Figure 1 e for both deposition temperatures. First, LSM electrodes with narrow grains (LSM_600_) exhibit a much higher current density for the same applied voltage and the same experimental conditions than an electrode with wider grains (LSM_900_). Thus, the total electrode polarization resistance varies in accordance with the grain size. This can be understood from a previous study on very similar LSM films with thermally driven ^18^O depth profiles.5f There, it was found that the diffusion in LSM grain boundaries was approximately three orders of magnitude faster than that in LSM grains and also that surface oxygen exchange coefficients were much larger for grain boundaries; the more grain boundaries the higher the effective oxygen exchange rate of the film. Therefore, the current is higher for films with small grains.

Second, the different grain widths affect the shape of the current decay. Irrespective of the applied bias, the measured current response of all microelectrodes deposited at 600 °C shows an exponential decay function (Figure 1 e, —). The current microelectrodes deposited at 900 °C exhibit additional time‐dependent features (Figure 1 e, —): a fast current decrease is followed by an increase and a plateau. This indicates that at least two processes with different time constants take place, possibly stoichiometry polarization of grains and grain boundaries. A more detailed interpretation is beyond the scope of this paper; here, we only conclude that the microstructure strongly affects the electrochemical properties of LSM films.

**Figure 2:**
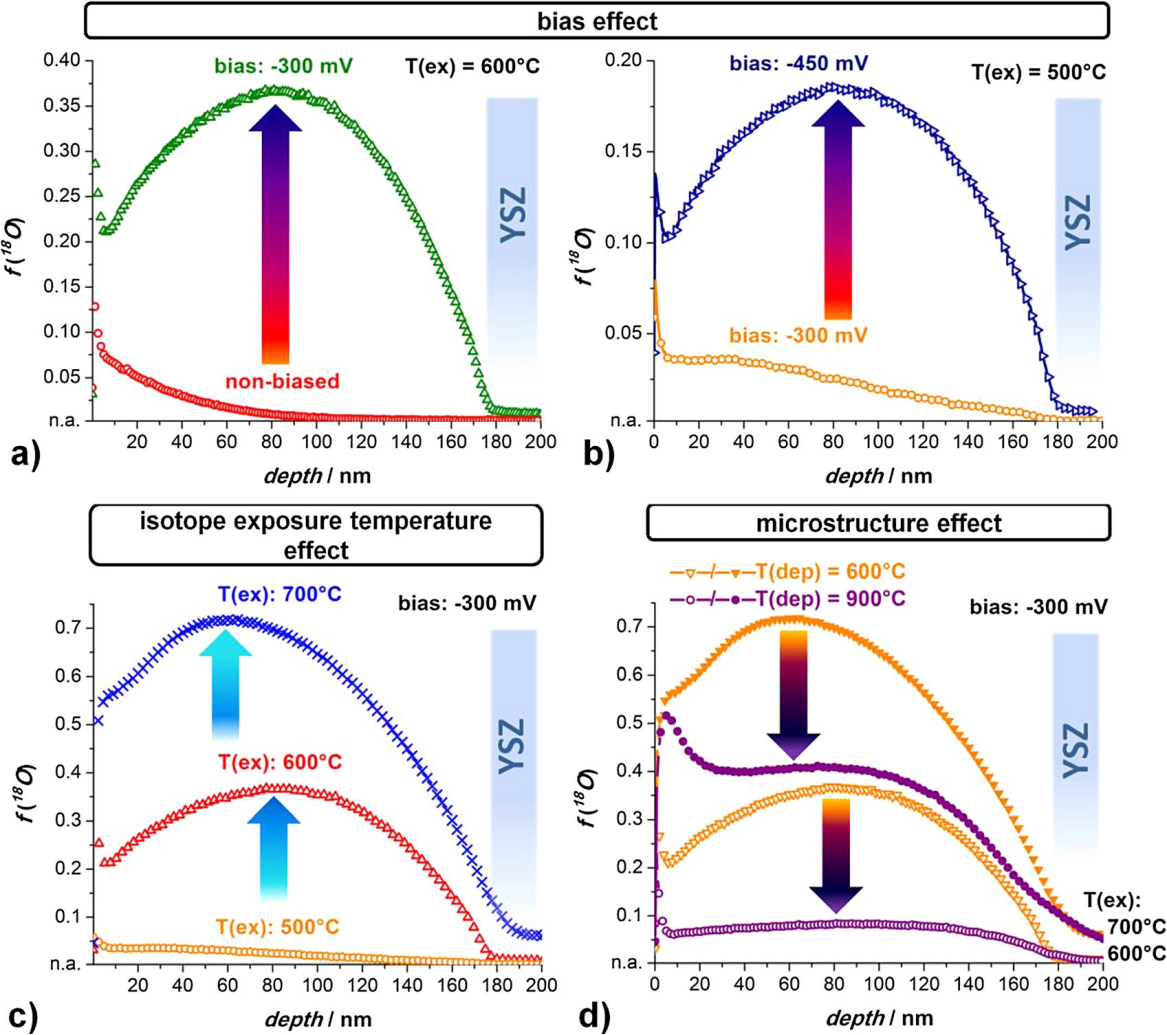
a) ^18^O tracer depth profiles in columnar LSM films (LSM_600_) resulting without bias (○) and with applied cathodic bias (−300 mV) (**▵**, El1). b) Isotope depth profiles measured on microstructurally identical layers (LSM_600_) but with different applied cathodic bias (El3: −300 mV and El4: −450 mV). c) The tracer measurements on structurally identical layers with the bias (−300 mV) applied at different temperatures (El3: 500 °C, El1: 600 °C, and El2: 700 °C). d) The effect of the LSM microstructure for the same bias (−300 mV) but different tracer exposure temperatures: 600 and 700 °C (n.a.=natural abundance = 0.00205).

A typical tracer depth profile obtained on LSM electrodes without applied bias consists of two parts: a steep near‐surface decrease in the ^18^O concentration that is followed by a shallow decay of isotope fraction (Figure 2 a, ○). In accordance with Ref. 5f, this indicates two parallel diffusion processes: The first profile part is dominated by diffusion in the bulk of the LSM grains. The following long tail up to the LSM/YSZ interface is caused by fast grain boundary diffusion and continuous tracer “leakage” into the bulk, compare type B diffusion in Harrison's classification of grain boundary diffusion.^8^ The concentration at the LSM/YSZ interface is above the natural abundance of ^18^O, and a very shallow profile is visible in YSZ, which indicates very fast diffusion in YSZ. The tracer ions in YSZ are more relevant for proper data analysis than one might expect from their low level, as their total amount in the 500 μm thick YSZ single crystal can become quite significant.

The ^18^O depth profile of a polarized LSM microelectrode is shown in Figure 2 a (**▵**). After applying a cathodic bias of −300 mV at 600 °C for 10 min to a LSM microelectrode (El1), the ^18^O fraction in the film is strongly enhanced. In the first few nanometers, the profile shapes look similar for both types of tracer experiments, with −300 mV and without bias (Figure 2 a). However, at a depth of around 5–10 nm, the depth profile shows an apparent bias‐induced uphill diffusion with increasing ^18^O concentration, which even rises above the surface concentration level. The ^18^O fraction reaches a maximum at roughly the center of the film and decreases towards the LSM/YSZ interface. The ^18^O fraction at this interface is significantly increased from 0.28 % for the nonbiased sample to 1 % for the cathodically biased sample.

To investigate whether the uphill diffusion shape and the high absolute isotope level were indeed caused by an applied cathodic bias, isotope incorporation experiments were performed at a lower temperature (*T*_ex_=500 °C) on microstructurally identical LSM layers by applying cathodic biases of −300 and −450 mV. The resulting tracer profiles under different cathodic biases are shown in Figure 2 b. Higher cathodic bias leads to substantially higher tracer fractions in LSM and an increase of the near surface tracer fraction from 7.5 to 14 %. The uphill profile is very pronounced for −450 mV with a maximum at a depth of 85 nm but transforms into a kind of plateau (up to 40 nm depth) for −300 mV.

The effect of the diffusion temperature is shown in Figure 2 c for LSM electrodes exposed to ^18^O_2_ between 500 and 700 °C. The results obtained at higher temperatures indicate a very pronounced “uphill diffusion”. Given that the depth of the peak value shifts towards the surface, from about 80 nm depth at 600 °C to roughly 60 nm depth at 700 °C, the slope of this uphill part is even larger at 700 °C at −300 mV. Furthermore, the first steep tracer decrease, which for thermal oxygen diffusion is attributed to bulk diffusion, is disguised at the higher temperature by the uphill profile.

The clearest indication of the mechanism behind the unusual diffusion profiles comes from isotope incorporation experiments performed on microstructurally different LSM_600_ and LSM_900_ layers with a cathodic bias of −300 mV at temperatures of 600 and 700 °C. Changing from a LSM_600_ (Figure 2 d, violet symbols) to a LSM_900_ (Figure 2 d orange symbols) microelectrode with much larger grains leads to a drastic change in the depth profile. At 600 °C, the near surface feature is essentially the same for both LSM microelectrodes. The “uphill diffusion” part with its maximum near the center of the LSM film, however, is much less pronounced for LSM_900_ than it is for the electrode with the small grains (LSM_600_). Also, the overall isotope level is much lower in LSM with a lower density of grain boundaries. This strongly suggests a crucial role of grain boundary diffusion.

## 3 Discussion

**Figure 3:**
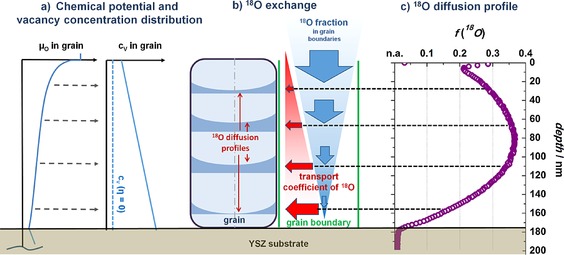
a) Sketch of the local oxygen chemical potential *μ*_O_ in the LSM/YSZ system, with a step function at the surface induced by the surface incorporation resistance and a decay in the LSM film caused by diffusion limitation; this leads to the sketched oxygen vacancy concentration profile. b) Illustration of the processes leading to apparent uphill diffusion in the measured ^18^O depth profiles: depth‐dependent decrease in the ^18^O concentration in grain boundaries (blue arrow) and increasing capability for in‐plane ^18^O transport in grains due to higher oxygen vacancy concentration (red arrow). This leads to the depth‐dependent in‐plane ^18^O profiles sketched. c) Measured ^18^O tracer profile in LSM thin film obtained at 600 °C with −300 mV applied bias.

To explain the diffusion profiles, particularly their unusual shapes with apparent uphill diffusion, we have to discuss the defect chemical effects occurring after applying a bias voltage. First, we consider the changes in the bulk of a mixed conducting electrode upon polarization. The overpotential *η* leads to a spatially varying chemical potential of oxygen *μ*_O_. The two extreme cases are given by oxygen reduction by a bulk path with rate‐limiting surface kinetics (e.g. found for Sr‐doped LaCoO_3−*δ*_ electrodes)^9^ and a bulk path with rate‐limiting ion transport. In the first case, polarization leads to a step in *μ*_O_ at the surface and a constant *μ*_O_ in the electrode. In the second case, the situation corresponds to Wagner–Hebb polarization^10^ with electron blocking at the electrode/YSZ interface. Hence, the chemical potential of oxygen varies within the electrode. From earlier tracer and impedance measurements^11^ we know that for LSM both surface exchange kinetics and oxygen bulk transport are relevant, and thus, a chemical potential distribution with surface step and bulk decay results (see Figure 3 a). Assuming negligible changes in the electronic majority charge‐carrier concentration and, thus, a rather constant and polarization‐independent chemical potential of electrons (*μ*_e_), we get from *μ*_O_+*μ*_V_+2 *μ*_e_=0 (*μ*_V_=chemical potential of oxygen vacancies) [Eq. (1)]:

(1) $$\nabla \mu_{0}\approx -\nabla \mu_{V}$$

For the small oxygen vacancy concentration *c*_V_ and a bulk concentration without bias (*c*_V_^0^), we thus find [Eq. (2)]:

(2) $$\nabla \mu_{0}=-RT\nabla \ln\frac{c_{V}}{c_{V}^{0}}=-\frac{RT}{c_{V}}\nabla c_{V}$$

in which *R* and *T* denote the gas constant and temperature, respectively. This means that upon bias, the oxygen vacancy concentration in LSM may drastically change. This can be quantified by considering the chemical permeability [Eq. (3)]:

(3) $$\sigma_{0}=\frac{\sigma_{\mathrm{ion}}\cdot \sigma_{\mathrm{eon}}}{\sigma_{\mathrm{ion}}+\sigma_{\mathrm{eon}}}\approx \sigma_{\mathrm{ion}}$$

with ionic and electronic conductivities *σ*_ion_, *σ*_eon_, that determine the vacancy flux density *J*_V_ by [Eq. (4)]:^12^

(4) $$J_{V}=\frac{\sigma_{O}\nabla \mu_{O}}{4F^{2}}\approx -\frac{\sigma_{\mathrm{ion}}}{4F^{2}}\cdot \frac{RT}{c_{V}}\cdot \nabla c_{V}=\frac{RT}{2F}\cdot u_{V}\cdot \nabla c_{V}=-D_{V}\cdot \nabla c_{V}$$

in which *D*_V_ and *u*_V_ are the vacancy diffusion coefficient and mobility, respectively, and *σ*_ion_=2*F*⋅*c*_V_⋅*u*_V_ (*F*=Faraday's constant).

In the steady state, *J*_V_ is constant, and a linear vacancy concentration profile with a higher vacancy concentration at the LSM/YSZ interface results (see Figure 3 a). Accordingly, the bulk tracer diffusion coefficient *D*_b_ also varies in the LSM film, as it is proportional to the oxygen vacancy concentration by [Eq. (5)]:

(5) $$D_{b}=f_{c}\cdot c_{V}\cdot D_{V}$$

with correlation factor *f*_c_.

This consideration of bulk defect chemistry upon polarization, together with fast oxide ion diffusion along grain boundaries, already qualitatively explains the observed profile shape. The local depth‐dependent chemical potential modifies the tracer bulk diffusion coefficient in LSM such that diffusion in the LSM grains is relatively slow close to the surface and becomes faster towards the LSM/YSZ interface. This affects the shape of the bulk diffusion profile (which is particularly relevant near the surface) but is even more important for “leakage” of the tracer from the fast grain boundaries into the grain. The in‐plane transport coefficient of a grain strongly increases with depth. This is indicated by red arrows in Figure 3 b. However, the driving force for tracer “leakage” from a fast grain boundary into the grain strongly depends on the tracer fraction in the grain boundary. Near the surface, the ^18^O concentration in the grain boundaries is highest, and the blue arrows in Figure 3 b indicate that the ^18^O fraction decreases with depth.

Qualitatively, the product of the transport coefficient (represented by red in‐plane arrows) and driving force (represented by blue across‐plane arrows) determines the resulting tracer fraction at a certain depth. Near the surface, a smaller fraction is incorporated from the grain boundaries into the grains due to the low bulk diffusion coefficient. The diffusion coefficient in the grains is strongly enhanced at some depths, and thus, more ^18^O is incorporated from the grain boundary into the grain. The tracer fraction in the grain boundary becomes low near the LSM/YSZ interface, and again, less tracer transfers from the grain boundary to the grain, despite the high bulk diffusion coefficient. This should lead to in‐plane isotope diffusion profiles in grains as sketched in Figure 3 b, and exact shapes are discussed in the finite element modeling part below. Our SIMS measurements cannot resolve the lateral (in‐plane) profiles within a single grain but integrate over many grains. In parallel to this grain boundary diffusion with leakage into the grain, across‐plane bulk diffusion originating at the surface takes place. Close to the surface this adds an additional tracer fraction with a sharp decay due to slow bulk diffusion.

Accordingly, we can expect exactly the profile shape found in the experiments (Figure 3 c): a sharp drop close to the surface and a maximum oxygen fraction at some depth due to very pronounced tracer “leakage”. The chemical potential variation sketched in Figure 3 a is expected to also vary the vacancy concentration and, thus, the tracer diffusion coefficient in the grain boundary. However, this should only modify the exact tracer concentration profile along the grain boundary, that is, the decay function of the driving force for tracer leakage (blue arrows in Figure 3 b), but does not alter the main considerations.

**Figure 4:**
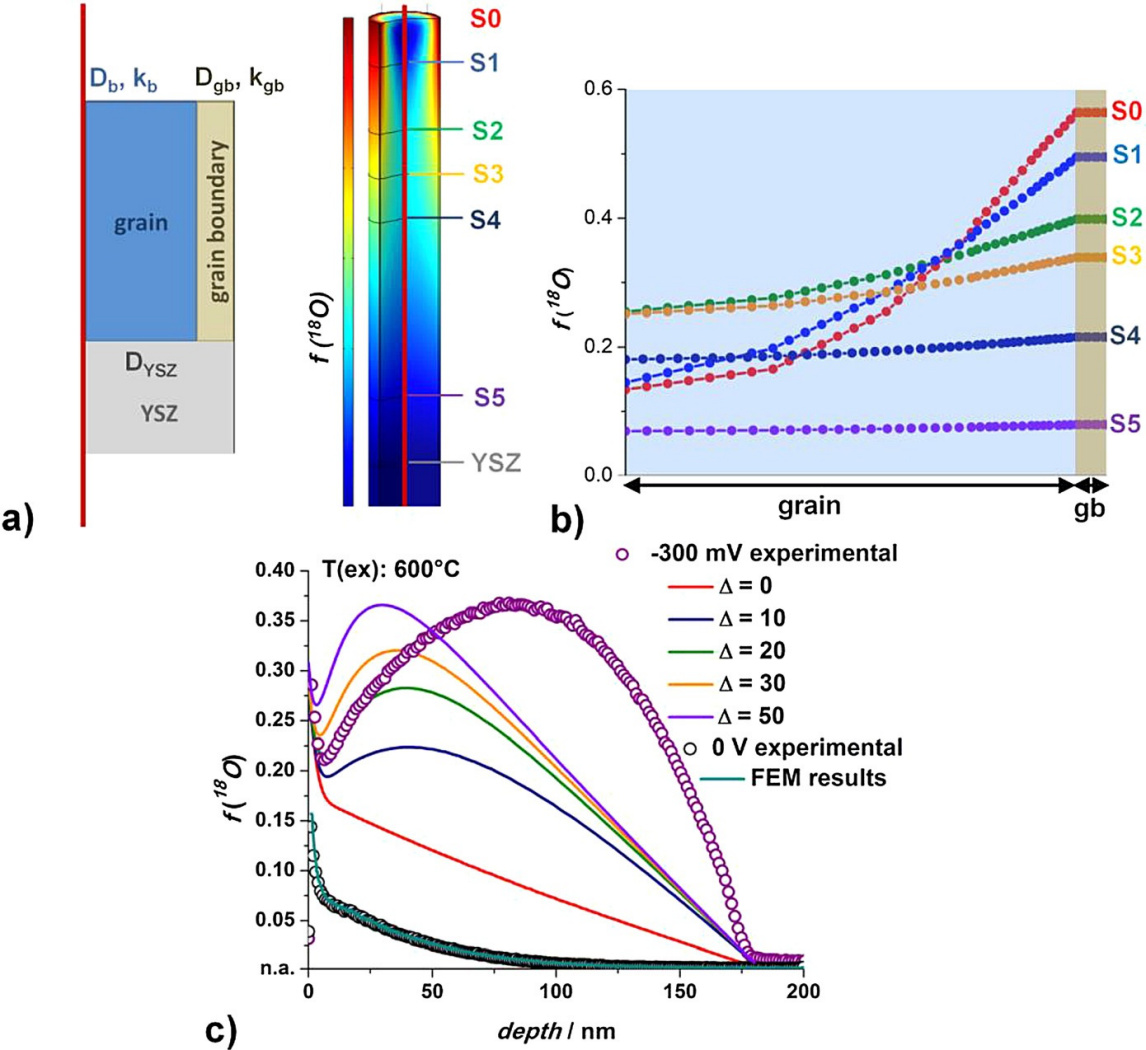
a) Sketch of the cylindrical finite element model that consists of three domains: LSM grain (defined by *D*_b_ and *k*_b_), grain boundary (defined by *D*_gb_ and *k*_gb_), and YSZ substrate (defined by *D*_YSZ_). *D*_b_ varies linearly from the surface to the LSM/YSZ interface, *D*_gb_ was constant. b) In‐plane tracer fraction profiles in different depths (S0 to S5, indicated in a) calculated for *Δ*=30. c) The experimental ^18^O isotope depth profiles of thermal oxygen diffusion at *T*_ex_=600 °C (○) and bias‐based transport at −300 mV (○). In the bias case, five solutions of the finite element (FEM) calculations are shown, for which *Δ* was varied from 0 to 50.

On the basis of these assumptions, profiles were also modeled by finite element calculations (COMSOL Multiphysics) for a cylindrically shaped grain. The model includes three domains representing diffusion in a grain (*D*_b_), along the grain boundary (*D*_gb_), and in the YSZ substrate (*D*_YSZ_). Diffusion coefficient values in YSZ (*D*_YSZ_) were taken from conductivity measurements. Moreover, two different oxygen surface exchange coefficients for grain (*k*_b_) and grain boundary (*k*_gb_) were considered in the model. A sketch of the model used for the calculations is shown Figure 4 a. As initial or boundary condition, the natural abundance in the sample was set to 0.00205 (given by the National Institute of Standards and Technology) for *t*=0 and the ^18^O fraction during the experiment was set to 97.1 % (as provided by tracer gas supplier).

First, modeling was performed to describe the measured thermal diffusion profiles; details are described in Ref. 5f. In this case, four individual parameters (*D*_b_≠*D*_gb_ and *k*_gb_≠*k*_b_, all without any depth dependence) allow successful fit to experimental tracer profiles (Figure 4 c, ○ and green line). To minimize unknown parameters, the mean grain width of such films was set to 30 nm for LSM_600_, and the width of grain boundaries in LSM was fixed to 2 nm. The fit parameters of a thermal isotope diffusion profile are listed in Table 2 and reveal diffusion (*D*) and surface exchange coefficients (*k*) for the grain boundaries (gb) that are about three orders of magnitude higher than those for the grains (b).

**Table 2:** Parameters for nonpolarized and polarized (−300 mV) LSM microelectrodes (El1, *T*_ex_=600 °C) used in the finite element calculations that are shown in Figure 4.

Bias [mV]	*D*_gb_ (*z*=0) [cm^2^s^−1^]	*k*_gb_ [cm s^−1^]	*D*_b_ (*z*=0) [cm^2^s^−1^]	*k*_b_ [cm s^−1^]	*Δ*
0	8.0×10^−14^	1.5×10^−8^	4.0×10^−17^	2.3×10^−11^	–
−300	2.3×10^−12^	2.0×10^−7^	1.6×10^−16^	8.5×10^−11^	0–50

Oxygen tracer motion in electrochemically polarized oxides consists of two flux terms; one that describes standard tracer diffusion (i.e. counter diffusion of ^18^O and ^16^O ions) and one that represents the unidirectional ionic current flux through the entire sample. A general discussion of this combination of fluxes is given in Ref. 12. There, it is shown that in the steady state with a current density *j* in the across‐plane (*z*) direction, Equation (6) has to be solved.

(6) $$\frac{\partial c_{{}^{18}O}}{\partial t}=\mathrm{div}\left(D\mathrm{grad}\left(c_{{}^{18}O}\right)\right)-\frac{j}{2Fc_{\mathrm{total}}}\frac{\partial c_{{}^{18}O}}{\partial z}$$

The symbols *c* ^18^O and *c*_total_ denote the ^18^O tracer and total oxide ion concentrations, respectively. In our case, we have to consider a spatially varying tracer diffusion coefficient *D* and different current densities in grain boundaries and grains. In Ref. 12 it is also discussed that the relevance of the second flux term in Equation (6) (proportional to *j*) scales with the ^18^O tracer traction and is small relative to the diffusional term for ^18^O fractions below 10 %.

It is beyond the scope of this paper to analyze our measurement data quantitatively, and thus, we restrict our modeling to the first (standard diffusion) term in Equation (6) even though tracer fractions in the 30 % range are found. Accordingly, for voltage‐driven tracer depth profiles the performed FEM calculations are similar to those without a current. Only spatial variation of the diffusion coefficient due to stoichiometry polarization is introduced. In accordance with Equation (4) and *J*_V_=constant (steady state), the bulk grain tracer diffusion coefficient varies linearly according to [Eq. (7)]:

(7) $$D_{b}\left(z\right)=D_{b}\left(z=0\right)\cdot \left(1+\Delta \frac{z}{h}\right)$$

in which *h* denotes the LSM film thickness (in this case −180 nm) and *Δ* represents the enhancement factor of the grain diffusion coefficient relative to the value at the surface [*D*_b_(*z*=0)]. Only voltage‐induced variation of the bulk diffusion coefficient *D*_b_ was assumed for simplicity; the (larger) grain boundary diffusion coefficient *D*_gb_ was still assumed to be depth independent in these calculations. The simulation was performed in a steplike process. The calculated profile was first adjusted to the steep near‐surface region of the exemplary measured data (LSM_600_, *T*_ex_=600 °C, −300 mV) by changing *D*_b_ and *k*_b_. Accordingly, this part of the profile was again attributed to slow oxygen incorporation into the grain and slow oxygen diffusion. The values of *D*_gb_ and *k*_gb_ were then chosen to reach the measured ^18^O concentration level at the LSM/YSZ interface. At a depth of about 10 nm, at which the measured uphill diffusion starts, the grain boundary contribution becomes visible in the profile. This feature was finally adjusted by modifying the enhancement factor *Δ* as depicted in Figure 4 c.

In this manner, profiles with apparent uphill diffusion can easily be reproduced, and the calculations confirm our qualitative interpretation. The in‐plane isotope profiles obtained for *Δ*=30 are shown in Figure 4 b. Near the grain surface, a low value of *D*_b_ limits the diffusion from the grain boundary to the grain; therefore, “small” integrals of the isotope fraction are found for cross sections S0 and S1. At some depth, *D*_b_ becomes larger and more oxygen diffuses from the grain boundary into the grain (highest integrated amounts for S2 and S3). Approaching the interface, a high value of *D*_b_ is found but already much less isotope is available in the grain boundary (S4 and S5). Thus, in‐plane profiles become rather flat and include less tracer ions.

However, within the framework of the given model the exact shape and absolute height of the measured curve cannot be fitted accurately. Also, additional consideration of the depth dependence of *D*_gb_ is not sufficient for an accurate fit, as the second term in Equation (6) is neglected. Moreover, the current versus time measurements (Figure 1 e) already indicated that steady state is not established for a significant part of the overall diffusion time. This is an unavoidable consequence of the gas exchange process (see the Experimental Section). Hence, additional time dependencies and parameter modifications come into play: 1) Establishing the steady‐state profile of *μ*_O_ takes some time (given by chemical diffusion and chemical surface exchange coefficients). During this period, all parameters, that is, *k*_b_, *k*_gb_, *D*_b_(*z*), and *D*_gb_(*z*), are time dependent. 2) This time dependence most probably includes different timescales, as chemical diffusion is also expected to be faster along grain boundaries, and thus, stoichiometry polarization of the grains takes place not only from the surface (in the *z* direction) but also from the grain boundaries with temporal in‐plane vacancy concentration variation.

Implementing all these additional aspects into the model would be required to finally quantify the measured profiles and to deduce information on the exact bias dependence of the *k* values and the contribution of *Δ*. The latter also indicates how much of the driving force *η* is reflected by a surface step of *μ*_O_ and by $\nabla \mu_{O}$ in the grain. This detailed analysis is beyond the scope of this paper and requires further experimentation. However, we may still discuss the parameters found for the very qualitative “fit procedure” done so far (Table 2). All parameters are enhanced upon bias voltage, that is, the *k* factors of the bulk and grain boundaries are substantially larger and also the *D*(*z*=0) value in the grain is higher than that for thermal diffusion. Hence, a certain step of *μ*_O_ at the surface is present, which indicates combined surface/transport rate limitation. According to the preliminary fit, concentration enhancement factors of several tens are most realistic for −300 mV. This would also be in agreement with the upper limit of the *Δ* value (*Δ*_max_) realized for a *μ*_O_ curve without surface step, that is [Eq. (8)]:

(8) $$\eta =\frac{RT}{2F}\cdot \ln\Delta$$

and thus Δmax=e2Fη2FηRTRT=2901 for −300 mV at 600 °C.

## 4 Conclusions

Defect chemical processes and ion transport in polarized LSM microelectrodes were investigated by means of voltage‐driven ^18^O tracer gas incorporation and subsequent SIMS analysis. The measured dc current was enhanced upon reducing the LSM grain size and thereby upon increasing the contribution of grain boundaries to oxygen reduction. Oxygen isotope depth profiles of voltage‐driven ^18^O incorporation were characterized by very uncommon uphill‐like diffusion with a ^18^O tracer maximum in the center of the LSM film. This effect was caused by the interplay of fast oxide ion diffusion along grain boundaries and stoichiometry polarization of LSM upon application of a voltage, which led to a vacancy concentration gradient in the LSM grains. This was particularly pronounced for LSM films with small grains. Numerical finite element simulations confirmed that oxygen transport in two parallel paths, that is, by grains and grain boundaries, can lead to apparent uphill diffusion profiles. Preliminary quantitative analysis indicates that surface incorporation kinetics and diffusion both contribute to the rate limitation in polarized LSM microelectrodes and that both are accelerated by an applied cathodic bias in grains as well as in grain boundaries.

## Experimental Section

### LSM Thin Film Deposition

Columnar LSM thin films were prepared by pulsed laser deposition (PLD). The PLD target was made from La_0.8_Sr_0.2_MnO_3_ powder (Sigma–Aldrich, USA), which was isostatically pressed and sintered for 12 h at 1200 °C in air. Thin LSM films were deposited on polished YSZ (100) single crystals (9.5 mol % Y_2_O_3_, CrysTec GmbH, Germany) by using a KrF excimer laser (*λ*=248 nm, COMPex Pro 101 F, Lambda Physics, Germany). Laser beam energy was set to 400 mJ per pulse at 10 Hz pulse frequency. The deposition was performed under an O_2_ atmosphere (4 Pa) and with a target–substrate distance of 6 cm. To vary the microstructure of the LSM layer, two deposition temperatures (*T*_dep_) of 600 °C (LSM_600_) and 900 °C (LSM_900_) were used, which was monitored by a pyrometer (Heitronics KT‐19.99, Germany). The film thickness was controlled by a known deposition rate. Squared 490×490 μm^2^ or 390×390 μm^2^ LSM microelectrodes were prepared from these films by UV photolithography and chemical etching in concentrated hydrochloric acid. A platinum counter electrode was brushed on the back side of the YSZ substrate. The grain widths and surface topography of the LSM thin films was checked by atomic force microscopy (AFM, Nanoscope V, Bruker Nano). Samples with microelectrodes were divided into smaller pieces, which thus allowed several samples with the same LSM film thickness and microstructure to be investigated.

### Oxygen Tracer Incorporation upon Cathodic Polarization and Profile Analysis

A LSM microelectrode was contacted by a Pt/Ir tip (see Figure 1), and the quartz tube with the sample was moved into a tube furnace.^7^ After thermally equilibrating this gas‐exchange set up in air at temperatures (*T*_ex_) of 500, 600, and 700 °C, the system was evacuated to a pressure of roughly 1 Pa. An ^18^O_2_ tracer gas atmosphere (200 hPa, 97.1 %, Campro Scientific, Germany) was then filled into the sample chamber and immediately a cathodic bias of −300 or −450 mV was applied to the contacted LSM microelectrode by means of a POT/GAL 30V 2A test interface together with an Alpha‐A High Resolution Dielectric Analyzer (both Novocontrol, Germany) in a dc mode (software WINCHEM and WINDETA Novocontrol, Germany). The dc current was monitored during the entire experiment. Given that each sample had numerous microelectrodes, unpolarized LSM was also exposed to ^18^O_2_, which allowed comparison with thermal diffusion. The ^18^O fraction in the gas‐exchange chamber was checked by a mass spectrometer (Pfeiffer GSD320 with QMG 220, Germany) and was in agreement with the ^18^O fraction given by the supplier. The cathodic overpotential *η* of the microelectrodes was determined by subtracting the voltage drop in the electrolyte from the applied bias voltage *U*. The electrolyte resistance was measured by impedance spectroscopy; the much smaller overpotential of the large counter electrode was neglected. All bias voltages and overpotential values are summarized in Table 1; in the text and figures, only the total bias *U* is indicated. Isotope incorporation experiments lasted 10 min, and afterwards, the quartz tube was moved out of the tube furnace and the sample was quenched under an ^18^O_2_ atmosphere (cooling rate: 100 °C min^−1^).

A pre‐annealing step of the thin films prior to the isotope experiments would be beneficial to chemically equilibrate LSM and thus to avoid chemical diffusion.5e, 13 Also, establishment of a current steady state prior to tracer exposure would be helpful if a bias voltage is applied. However, in our experiments neither pre‐equilibration nor steady state was possible, as the gas switch from ambient air to oxygen isotope gas required evacuation of the exchange chamber. This step annihilates any chemical pre‐equilibration or steady state. Gas exchange at room temperature was also not an alternative due to the very short tracer exposure times needed for thin films and the finite heat‐up time. Hence, a contribution of chemical diffusion to the tracer experiment could not be avoided.

The resulting ^18^O depth profiles were subsequently investigated by time‐of‐flight secondary ion mass spectrometry (ToF‐SIMS 5, ION‐TOF GmbH, Germany). Measurements were done in the collimated burst alignment (CBA) mode with Bi_3_^++^ primary ions (25 keV). This mode allowed accurate determination of ^18^O fractions over a broad intensity range.^14^ Negative secondary ions were analyzed in an area of 45×45 μm^2^. For the sputtering of material, 2 keV Cs^+^ ions were applied with a sputter crater of 350×350 μm^2^ and sputtering ion current of 120 nA. Surface charging was compensated with an electron flood gun. The tracer fraction *f*(^18^O) was obtained by normalizing integrated intensities *I* of ^18^O and ^16^O in the mass spectra according to Equation (9):

(9) $$f\left({}^{18}O\right)=\frac{I\left({}^{18}O\right)}{I\left({}^{16}O\right)+\left({}^{18}O\right)}$$

The sputtering rate of LSM thin films was determined from the depth of a sputtered crater that was measured by digital holographic microscopy (DHM, Lyncee Tec, Switzerland). The isotope depth profile measurements were performed on biased and on nonbiased microelectrodes, which thus allowed thermally and bias‐driven oxygen tracer diffusion to be probed.
